# Exploring the Animal Models of Gastrointestinal and Emotional Comorbidity

**DOI:** 10.1002/brb3.71135

**Published:** 2025-12-17

**Authors:** Yan‐hui Wang, Xiang‐rui Kong, Xin‐zhu Ye, Su Pu, Xin‐xin Ren, Qing‐yang Huang, Ya‐qing Lu, Hao Wang

**Affiliations:** ^1^ College of Integrated Chinese and Western Medicine (School of Life Sciences) Anhui University of Traditional Chinese Medicine Hefei Anhui China; ^2^ College of Graduate School Anhui University of Traditional Chinese Medicine Hefei Anhui China; ^3^ Anhui Province Key Laboratory of Meridian Viscera Correlation Ship Anhui University of Chinese Medicine Hefei Anhui China

**Keywords:** anxiety and depression, comorbidity model, functional dyspepsia, modeling methods

## Abstract

**Background:**

With the comorbid anxiety/depression in gastrointestinal diseases occur at a high rate worldwide, it is particularly important to discover and optimize the protocols of comorbidity models, thus further to reveal the mechanism and develop the treatment strategies of comorbidity.

**Methods:**

Using “functional dyspepsia (FD), animal model, anxiety, depression” as search terms, this search is conducted in PubMed, Web of Science, and SpringerLink databases, with the search period from 2020 to 2025.

**Results:**

We find that both iodoacetamide (IA) gavage and tail clamping (TC) are classical methods for establishing FD model; physical stress such as “ice water gavage” or “irregular feeding (IF)” also used. We propose that IA + TC + IF or TC + IF + ice water gavage can be used to prepare comorbidity models in future. Maternal separation (MS) may also potentially become one of the effective models for studying comorbidities. In anxiety/depression model, we show that both chronic restraint stress (CRS) and chronic unpredictable mild stress (CUMS) are classical models for emotional disorders. We suggest CRS/CUMS combines with IA/IF/TC can better to establish gastrointestinal‐emotional comorbidity model which through brain–gut cross talk. Then the review also develops a comprehensive and standardized evaluation system for comorbidity models, with recording behavioral indicators related to gastrointestinal/emotional disorders, while including multidimensional parameters such as the microbiota, neuroinflammation, metabolomics, brain–gut peptide, and neurotransmitters.

**Conclusion:**

This study is the first comprehensive comparison of FD and anxiety–depression modeling protocols to propose effective protocols of comorbidity models and develop a comprehensive and standardized evaluation system for comorbidity models.

## Graphical abstract

In our review, we compare the modeling protocols of functional dyspepsia and anxiety/depression, proposing effective protocols of comorbidity models, such as IA + TC + IF, TC + IF + °C water gavage, MS, and CUMS/CRS + TC/IA/IF, and exploring the possible pathological mechanisms of comorbidity which may involve gut microbiota, microbial metabolome, immune activation, inflammation, neuronal activity, and neurotransmitter.

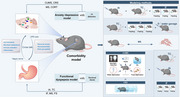

## 1 Introduction

With the acceleration of the pace of life and the increasing of work pressure, functional gastrointestinal disorders (FGIDs) have become serious health issues in modern society. FGIDs are caused by physiological, psychological, and social factors, and functional dyspepsia (FD) and irritable bowel syndrome (IBS) are common. An important feature of FGIDs is the comorbidity of emotional disorders, such as anxiety and depression. The clinical symptoms and pathological mechanisms of FGIDs and emotional disorders influenced bidirectionally (Ruan et al. 2023). It can be seen that FGIDs are cross‐organ diseases with dysfunction of the digestive system and central nervous system. FGIDs have been reclassified as disorders of gut–brain interaction (DGBI), reflecting the critical role of bidirectional communication between the gut and brain in these diseases and their impact on psychological comorbidity (Kraimi et al. 2024), given the emerging evidences linking gut microbiota, microbial metabolome, immune activation, and stress‐related neurocircuitry in DGBI (Person and Keefer 2021; Margolis et al. 2021). For example, neurons and glial cells in the enteric nervous system participate in intestinal immunity through gut–brain axis (GBA) to affect central nervous system (Agirman et al. 2021). The gut microbiota can modulate the gut barriers, there by constituting an important channel of communication across the GBA (Aburto and Cryan 2024). The microbial pattern flagellin can modulate vagal nodose neurons to affect brain activity (Liu et al. 2025). Therefore, the establishment of comorbidity models based on the “gut–brain interaction” has an important significance, which may provide “precise targets” for clinical interventions.

**FIGURE 1:**
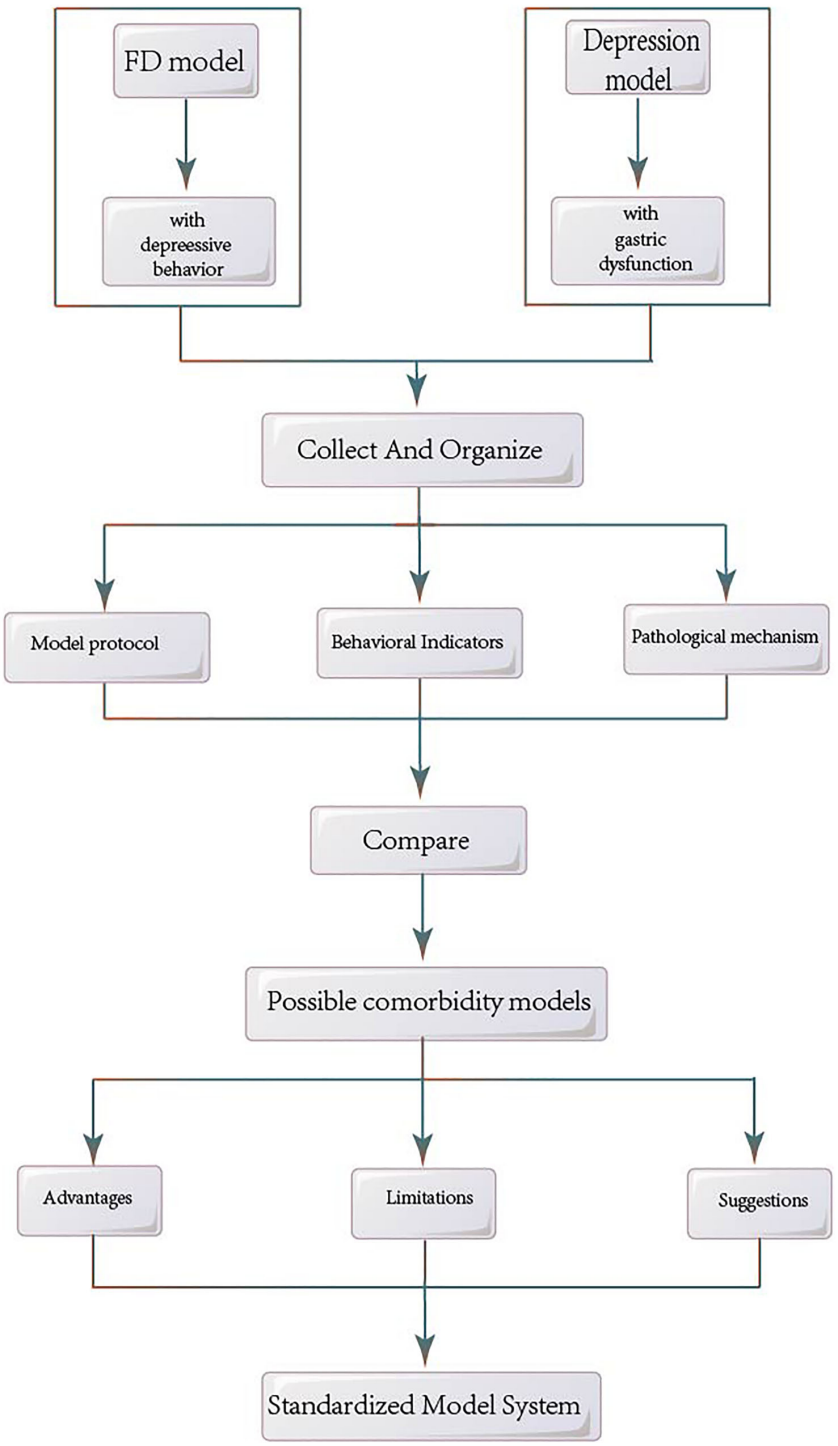
The flowchart of the study. FD, functional dyspepsia.

Animal models have been extensively used to explore pathological mechanisms and to develop new therapies for human diseases including FD and emotional disorders. During the modeling process of FD, in addition to exhibiting FD symptoms, the model animals also exhibit anxiety/depression behavior. In clinical practice, it has been found that FD patients often have psychiatric comorbidity, such as anxiety and depression (Cordner et al. 2021). In addition, the prevalence of depression or anxiety is higher in patients with FD compared to healthy people (Esterita et al. 2021). Similarly, negative emotions can also induce further development of FD. The clinical correlation between the FD and emotional disorders has attracted widespread attention of scholars. Although at present, separate models have been developed for FD (such as chemical method) and anxiety–depression (such as restraint stress). However, models simultaneously simulate both gastrointestinal and emotional disorders remain limited. The clinical essence of “bidirectional interaction” also cannot be replicated. The review aims to systematically compare FD models and anxiety/depression models to guide research of comorbidity models. Therefore, this study collects, organize, and compare literatures related to FD and anxiety–depression models from the past 5 years to find an effective protocols of possible comorbidity models from the characteristics of animal behavior, modeling methods, behavioral indicators, pathological mechanisms, and other aspects. And then we analyze the advantages and disadvantages of the possible comorbidity model in order to establish a standardized system of comorbidity models (shown as Figure 1).

## 2 Research Methods

The search is conducted in PubMed, Web of Science, and SpringerLink databases, with the search period from 2020 to 2025. Search terms include “functional dyspepsia, animal model, anxiety, depression.” The inclusion criteria for the literature: (1) published in the last 5 years (2020–2025); (2) animal models of rats or mice; (3) using effective experiments to test the efficacy of treatment for depression, anxiety, and/or FD; (4) measuring one or more cytokines, serum molecules, related protein molecules, mitochondria, mRNA molecules, neurotransmitters, and so forth, to study gastrointestinal function; (5) Behavioral tests to detect emotional changes. Exclude the following articles: (1) duplicate articles; (2) small reviews, systematic reviews, or meta‐analyses; (3) nonspecific articles, including the biological mechanisms of psychotropic drugs, FD overviews, and GBA overviews; (4) other mental disorders besides depression or anxiety; (5) commentaries or opinions. The literature search 70 articles which are included in this review.

## 3 Experimental Animals of FD/Anxiety–Depression Model

The FD model animals included in this study are primarily rats, especially SD rats, not Wistar rats. It may be related to the fact that Wistar rats are more easily influenced by stress factors, making them more sensitive to gastrointestinal function and anxiety–depression (Accarie and Vanuytsel 2020). However, in recent years, there has been an increase in using mice for modeling (Pan et al. 2024), which may be related to experimental studies involving gene mice or viral expression, and so forth (Dong et al. 2023). Therefore, although rats are predominantly used in FD model, it is also important to explore the methods for FD mice models to meet the needs of various experimental studies.

The anxiety and depression model animals included in this study are primarily mice. Some studies have found that various manipulation techniques can be better applied to mice. Moreover, research has found that the ability of mice to form long term contextual fear memories is stronger than rats (Akers et al. 2012). In the preparation of model, the mice are easier to induce anxiety and depression behaviors, and the effects of the same antidepressant drugs are different between rats and mice (Zhou et al. 2021). It is suggested that mice have advantages in the preparation of anxiety and depression models. However, some experiments also use rats for modeling. Research has found that rats exhibit stronger social behaviors, whereas mice exhibit weaker social behaviors (Żakowski 2020). Social behavior has an impact on the emotional changes in animals. Therefore, scholars should select appropriate species according to the experimental objectives.

Current preclinical research on the comorbidity of FD with anxiety and depression exhibits a significant gender imbalance. Most experiments use male animals primarily, which is significantly inconsistent with the reality that women have a higher prevalence of the diseases in clinical practice. Recent studies have focused on potential gender‐specific mechanisms, 5‐min forced swimming (FS) stress is a useful paradigm to study the female not male biased susceptibility to anxiety, this is because of CRFR1‐mediated hyperexcitation of oval nucleus of bed nuclei of the stria terminalis (ovBNST) CRF neurons in female mice encoding the female‐biased susceptibility to anxiety (Zhang et al. 2024). Chronic restraint stress (CRS) + chronic and unpredictable mild stress (CUMS) induced anxiety‐like behavior in female mice and depression‐like behavior in male mice. Sex differences in behavior may be related to hyperactivity of hypothalamic–pituitary–adrenal (HPA) axis, monoamine neurotransmitters, gut microbiota, inflammatory factors, and brain–gut metabolism (Qiao et al. 2025). Some studies even indicate that maternal separation (MS) exacerbated colonic sensitivity and mast cell and eosinophil infiltration in only females (Accarie et al. 2022). These findings highlight the importance of systematically incorporating female animals into disease modeling and studying gender‐specific mechanisms, thus to improve the clinical translation value of basic research.

## 4 FD Models With Secondary Emotional Symptoms (Shown as Table 1)

### 4.1 Iodoacetamide (IA) Gavage

In the single‐factor modeling experiments, IA gavage was used most. IA is a protease inhibitor that can induce mild gastritis in neonatal rats. The gastrointestinal function of newborns is fragile. During the period, stimulating the gastrointestinal tract with IA can affect its function in adulthood (Wu et al. 2021), such as motor dysfunction and damaged accommodation in adults (Zou et al. 2020; Ouyang et al. 2020), and even IA can influence brain emotions through the HPA axis (Liu et al. 2024). The rats were selected 10 days of age and gavaged with 0.2 mL 0.1% IA for 6/7 days. The FD model induced by IA gavage, which has a similarity with human FD patients (Guo and Gharibani 2024). Overall, the IA gavage is regarded as a classical chemical method for building FD model. Single‐factor modeling has certain limitations. Although the IA gavage causes gastric hypersensitivity, but the stress is temporary and the impact on gastrointestinal function in the later stages is not significant. So, most experiments selected multifactorial stimulation. Of them the IA gavage combined with tail clamping (TC) is used most. Rats were continuously IA gavaged for 6 days from the 10th day of born, on the 7 weeks of age, the stimulation of TC for 7 days (Han et al. 2021) or 9 days (Chen et al. 2023);or on the 10 weeks of age, TC stimulating for 14 days (Dong et al. 2022). The improved model can better simulate the pathogenesis of FD patients. It is consistent with the understanding of the disease in traditional Chinese medicine (TCM). According to TCM, “spleen deficiency and qi stagnation” is the most common syndrome of FD. Wu, Lu, et al. (2020) used IA‐lavage to mimic the trigger factor of spleen‐deficiency and TC to mimic the trigger factor of qi‐stagnation, building the FD model of “spleen‐deficiency and qi‐stagnation.” Modern medical research has shown that IA can induce motor dysfunction, visceral hypersensitivity, and gastrointestinal inflammation (Han et al. 2022). TC can lead to emotional disorders such as anxiety and depression in animals (Hou et al. 2022). The emotional and visceral sensations may through the brain–gut axis influence each other (Zhu et al. 2020). Therefore, IA gastric lavage combined with TC holds promise as an effective approach for establishing FD and emotional comorbidity models.

Besides, IA combined with modified small platform method (MSPM) induced FD model (Xiao et al. 2023). One study showed that the gastrointestinal motility disorder in the model may be related to PINK1/Parkin‐mediated mitophagy and division (Zhang et al. 2022). Furthermore, the FD model appeared serum levels of TNF‐α, and IFN‐λ increased, the gastric emptying delayed, the sucrose preference decreased, and the intestinal microflora disturbance (Bai et al. 2023). Therefore, this modeling method primarily induces FD gastrointestinal dysfunction and/or associated depressive behaviors. It may serve as a reference model for the comorbidity of FD with anxiety and depression.

### 4.2 Tail‐clip (TC) Stimulation

In the single‐factor modeling, the stimulation of TC has also been used frequently. Researchers used sponge clamp or surgical plier to clinch the distal end of the rats’ tails, three times a day, sustaining for 7 days (Song et al. 2020). Stress is an important factor in causing FD. TC irritates was intended to mimic the characteristics and elicit the symptoms of FD by inducing stress, as behaviors of stress (mood swings, depression, and anxiety) have been identified as strong factors in FD (Tang et al. 2020). It is closer to the pathogenic factors of human FD. The method is conformed to the syndrome of liver “qi” stagnation in TCM (Wang et al. 2024). Currently, clinical treatment of FD not only needs the drugs of regulating gastrointestinal motility disorders and visceral sensitivity but also needs the drugs of improving anxiety and depression (Zeng et al. 2024). Some researchers found that the stimulation of TC increased the activity of sympathetic nerve, enhancing the release of norepinephrine (NE), and upregulating the expression of β‐adrenergic receptors, which may be partially involved in the pathogenesis of gastric motility disorders (GMDs) in FD rats (Song et al. 2020). At the same time, the activity of the sympathetic nervous system is closely related to emotional regulation. Therefore, it is important to study the establishment of the comorbidity model from the effect of sympathetic nerve activity with stimulation of TC.

The interventions of multifactors can simulate various stimulation during the growth of human, which simulated the pathogenic factors of FD from different aspects. Among them, TC combined with irregular feeding (IF) is widely used. Wu, Lai, et al. (2020) established FD model with TC and IF, the rats appeared sluggish, exhausted, irritable, tense, and uncomfortable, these symptoms matched with the same description of FD with liver‐depression and spleen‐deficiency syndrome. TC induces a chronic stress response from the physical and psychological stress, which is similar to psychosocial stress in FD patients. IF simulates the irregular eating in FD patients (Xiao et al. 2021). Chen et al. used a two‐stage multifactorial approach for modeling, such as IA + TC + IF. The first stage, they used IA gavage in neonatal rats for 6 days, and the second stage, they used TC stimulation combined with IF at 7‐week‐old rats for 14 days. The results showed that the rats exhibited anxiety‐like behaviors and gastrointestinal dysfunction (Chen et al. 2021). TC + IF + 0°C ice and water gavage persisted for 20 days, the FD rats showed reduced food and weight loss, and intestine mucosal barrier damage, which may be relate to the TLR4/NF‐κB p65 pathway (Wang et al. 2023). Interestingly, a study on CUMS‐induced depression model is associated with gut microbiota, TLR4/MyD88/NF‐κB signaling, and HPA‐axis (Zeng et al. 2025). These results indicate there is a common pathogenesis with immune–microbiota–neuroendocrine mechanism in FD and depression. The GBA, integrating endocrine, neural, and immune signals, enables multiple biological indicators within the body to exert bidirectional effects on gastrointestinal function and emotional states (Ye et al. 2024; Barilla et al. 2025). This also provides a theoretical basis for selecting indicators in comorbidity models.

Recently, some scholars focused on the central mechanism of FD. Zhang et al. used TC+ place in water +45‐degree cage tilt and social isolation for 14 days. The mice appeared gastric dysfunction, which were drived by 5‐hydroxytryptamine (5‐HT) dorsal raphe nucleus (DRN) → Ach DMV → stomach axis, thus providing insights into the circuit basis for brain regulation of the stomach (Dong et al. 2023). Additionally, functional magnetic resonance imaging (fMRI) showed that FD patients/FD rats significantly correlated with basolateral amygdala (BLA), insular, hippocampus, thalamus, dentate gyrus, and medial prefrontal cortex (mPFC) (Chen et al. 2021; Sun et al. 2021). These brain regions are also closely associated with anxiety and depression (Wang et al. 2021; Yan et al. 2024). These results indicate that gastric dysfunction and emotional abnormality affect the same neural nuclei in brain, which may serve as an experimental basis for preparing a model of gastrointestinal and emotional comorbidity.

### 4.3 Maternal Separation (MS)

MS model is an early life stress that can affect the development of the nervous and digestive systems in childhood, leading to duodenitis and gastrointestinal dysfunction in adulthood (Duan et al. 2022). Some studies selected MS to induce FD model. From the first day of born to the 10th day, newborn rats are separated from mother for 2 h each day. At the 7–8 weeks of age, it was found that the rats exhibited gastric hypersensitivity, indicating the successful preparation of the FD model (Duan et al. 2021). MS experiments not only play a role in constructing FD models but also provide guidance for establishing anxiety and depression models. Xiong et al. (2025) reported that offspring mice with MS experience (MS mice) exhibited an increase in CD4+ T cells in peripheral blood and a severe deficit in mPFC‐dependent cognitive functions such as anxiety/depression emotion regulation. CD4+ T cells, as the core of immune regulation, may initiate CNS inflammation (White et al. 2025). The mPFC, a key center for emotional regulation, can also influence gastrointestinal function through the brain–gut axis pathway. Wang et al. (2022) reported that MS can successfully induce anxiety‐like behavior in adolescent mice that continues through adulthood, further accompanied by abnormal expression of Dnmt3a in the cortex and hippocampus. Beyond its effects on offspring, the impact of MS on maternal behavior is also noteworthy. Research has shown that MS dams displayed slightly higher depressive and anxiety‐like behaviors (Noori et al. 2020). In summary, MS experiments play a crucial role in elucidating the mechanisms underlying the comorbidity of FD with anxiety and depression. Whether newborn mice or female mice, their reactions in the experiment are all noteworthy.

### 4.4 Forced Swimming (FS)

FS is not commonly used alone to prepare the FD model; instead, it is often combined with IF. Tu et al. (2022) used FS combined IF to build FD for 42 days, the rats exhibited gastric dysfunction and gastrointestinal mucosal damage, which were associated with imbalances in brain–gut peptides, neurotransmitters, and reduced immunity. On the basis, some experiments have also added other stimulating factors to achieve better modeling effects. For example, Zhang et al. (2020) used FS + IF combined with extract substance of rhubarb gavage for 14 days, and the rats showed a series of FD symptoms accompanied by fatigue. In addition, FS + IF also combined with IA gavage induces the release of inflammatory mediators and histamine in rats, activating mast cell signaling pathways (Bai et al. 2024). IA gavage induced gastrointestinal motility disorders and increased visceral sensitivity. FS and IF simulated clinical overexertion and irregular diet, respectively. So, the method is closer to the pathogenesis of human FD. However, further research and exploration are still needed for the development of FD and emotional comorbidity models.

**TABLE 1:** FD model with secondary emotional symptoms.

Methods	Animal species	Gastrointestinal function	Anxiety/Depressive behavior	Mechanism
Iodoacetamide (IA) gavage	SD rats	CD3+ and mast cells↑ the intestinal mucosal permeability↑ gastric emptying, impaired gastric accommodation and gastric sensitivity	—	Gastrointestinal inflammation. Ghrelin↓ (Wu et al. 2021; Zou et al. 2020)
	BALB‐C mice	Slight inflammation; high intestinal permeability; the induction of visceral hypersensitivity; microbial community diversity↓	—	Sensitization of visceral afferent fibers and alterations in the excitability of central neurons (Liu et al. 2024)
	SD rats	Gastric distension↑	Long‐lasting increase in anxiety‐ and depression‐like behaviors in rats, together with an enhanced sensitivity of the hypothalamic pituitary–adrenal axis to stress	Lower vagal efferent activity and higher sympathovagal ratio (Guo and Gharibani 2024)
IA+ tail clamping (TC)	SD rats	Gastric emptying↓; intestinal propulsion rate of the MG↓	OFT: distance and mobile time↓; improvement in immobile time. activity level↓; irritable mood	Visceral hypersensitivity (Chen et al. 2023)
IA+ modified multiple platform method (MMPM)	SD male	Gastric emptying rate↓; disturbance of the numbers and structures of intestinal flora components; visceral hypersensitivity; small intestinal propulsion rate↓	The sugar water preference rates; visceral hypersensitivity; small intestinal propulsion rate↓	The imbalance in the gut–brain axis, 5‐HT↑ (Xiao et al. 2023) gut and brain communicate through the enteric nervous system and the hypothalamic–pituitary–adrenal axis. ↑ (Bai et al. 2023)
Tail‐clamping (TC)	SD rats	Gastric emptying rate↓, small intestinal propulsion rate↓,; mitophagy; the number of neurons in the hypothalamus↓; infiltration of inflammatory cells	Depression‐like behaviors; fidgety	The hippocampus is damaged, a hyperactive HPA axis (Hou et al. 2022) ghrelin ↓, the brain–intestinal axis (Tang et al. 2020)
TC + IF + 0°C water gavage	SD rats	The duodenal mucosa showed sever damage; inflammatory response	Irritability	Duodenal mucosal barrier resulted in inflammation (Wang et al. 2023)
TC + IF	SD rats/Wistar rats	Changes of gut microbiota; gastrointestinal hormone↓; gastric residual rate↑; intestinal propulsion rate↓; pepsin excretion↓	Exhausted, sluggish, uncomfortable, irritable, and tense, reduced activity	Brain–gut peptides↓; para secretion of brain–gut peptides (Wu et al. 2020)
TC + IA + IF	SD rats	Body weight↓; food and water intake↓; gastric emptying rate↓	Irritable and anxious; infighting↑	The activation of S1 (Chen et al. 2021)
TC + IF	SD rats	Gastric residual rate↓	Reduced activity; listlessness	Dysfunction of the brain–gut axis (Dong et al. 2023)
Maternal separation (MS)	SD rats	Gastric hypersensitivity, activated eosinophils↑, visceral hypersensitivity reaction	—	The CNS and autonomic nervous system (Duan et al. 2022). Micro‐inflammation; basal HPA axis activity (Duan et al. 2021)
Forced swimming (FS) + IF	Wistar rats	Body weights↓, diarrhea, spleen and thymus indices↓, duodenum mucosal permeability intestinal mucosa inflammation	Reduced activity	The brain–gut interactions (Tu et al. 2022)
	SD rats	the gastrointestinal motility was reduced↓	Exhausted, sluggish	CPK, MTL, and GAS↓. (Zhang et al. 2020)
FS + IA + IF	SD rats	The visceral hypersensitivity Gastric emptying rate↓, inflammatory cells, small intestinal propulsion rate↓, the mucosal layer was swollen	—	Simulate clinical overexertion and irregular diet respectively (Bai et al. 2024)

Abbreviations: 5‐HT, 5‐hydroxytryptamine; CNS, central nervous system; CORT, corticosterone; CPK, creatine phosphokinase; DRD2, dopamine receptor D2; FD, functional dyspepsia; GAS, gastric acid secretion; HPA, hypothalamic–pituitary–adrenal axis; MTL, motilin; OFT, open field test; PAR2‐PKC, protease‐activated receptor 2‐protein kinase C; SD, Sprague‐Dawley.

## 5 Depression/Anxiety Model With Secondary GI Dysfunction (Shown as Table 2)

### 5.1 Corticosterone (CORT) Injection

CORT injection and IA gavage both belong to chemical methods for modeling. The CORT‐induced anxiety/depressive model is relatively stable. Mice are subcutaneously injected with CORT (dosage of 20 mg/kg/day) for 3 weeks. The CORT is dissolved in 0.9% NaCl which contained 0.1% dimethyl sulfoxide (DMSO). As a result, the mice appear depressive‐like behavior. CORT induces depressive‐like behaviors and neuronal damage and enhances the levels of monoamine neurotransmitters in the serum of mice. Even CORT induces neuronal damage in depression mice by microglia activation and pro‐inflammatory response (Bai et al. 2022). These neuroinflammatory responses also occur during gastrointestinal dysfunction in FD. Studies showed that CORT induced depression‐ and anxiety‐like behaviors, possibly by increasing brain levels of NE (Wu et al. 2023). In addition, NE can inhibit gastric motility by binding to its receptor. Cai et al. (2022) found that CORT injection combined with CRS for 21 days, induced anxiety and depression. As a diagnostic index of anxiety and depression, the study assessed GABA and 5‐HT levels, the results showed that the GABA and 5‐HT levels were significantly decreased, and appeared histopathological changes in the hippocampus CA3 region. However, it did not observe indicators related to gastrointestinal function. So, whether CORT injection combined with other factors could serve as an effective strategy for preparing models of FD and emotional comorbidity remains to be further explored.

### 5.2 Chronic Restraint Stress (CRS)

CRS is achieved by placing mouse in a 50‐mL syringe with holes for 6 h daily. The central nucleus of the amygdala (CeA), as an important center for emotional regulation, the abnormality of its activity may induce emotional disorders. Some research studies reported that CRS (20 min/day for 14 days) could induce anxiety‐like behavior, which might be accounted for a reduction in synaptic connectivity of the CeA (Moreno‐Martínez et al. 2022) or decreased hippocampal 5‐HT, GABA, and NE levels and increased serum CORT and corticotropin‐releasing hormone (CRH) levels (Yan et al. 2023). Some other research studies indicated that CRS (3 h/day for 21 days or 2 h/day for 14 days) induced depression‐like behavior, which might be due to the change in morphology and density of dendritic spines of neurons in mPFC (Tse et al. 2023), or inactivated glutamate neurons in paraventricular nucleus of thalamus (PVT) and PVT → nucleus accumbens (NAc) circuit (He et al. 2025), or suppressed autophagy mainly in the lateral habenula (LHb) (Yang et al. 2025). However, another study showed that CRS disrupted kynurenine (Kyn) metabolism and endocrine function along the GBA, accompanied by the disrupted homeostasis of certain microbiota, which collectively contributed to the development of depression‐like behavior (Deng et al. 2021). In conclusion, the mechanism of CRS‐induced anxiety/depression involving central neuronal activity, synaptic connectivity, neural circuit, neurotransmitter, autophagy, HPA axis, and microbial‐GBA, and partially of them even also participated in the pathological process of gastrointestinal diseases. Recently, some researchers used CRS combined with other factors to induce gastrointestinal disorders model. Chen et al. (2024) applied CRS combined with IA gavage to establish chronic non‐atrophic gastritis (CNAG) mice model. CNAG mice showed gastrointestinal inflammatory responses, which may be closely related to the imbalance of the gut microbiota and bile acid metabolism. We used CRS combined with IF for 21 days to induce GMD model. We found that stress increased hippocampus glutamate content (Wang et al. 2020), activated CeA GABAergic neurons and inhibited dorsal vagal complex neurons (Wang et al. 2023), or inhibited GABAergic neurons in the spinal dorsal horn and activated sympathetic nerves to drive gastric dysfunction (Zhang et al. 2025). It can be seen that the imbalance of vagus and sympathetic nerves is one of the mechanisms of gastric dysfunction. These studies make it possible to use CRS establishing gastrointestinal and emotional comorbidity model in future.

### 5.3 Chronic Unpredictable Mild Stress (CUMS)

It is common to use CUMS to build anxiety–depression model. Stimulation of long‐term stress has been recorded as an important cause of anxiety and depression. The process of CUMS was as follows. Animals were exposed to multi‐stimuli for 28 days. Multi‐stimuli included deprivation of food for 24 h; deprivation of water for 24 h; day and night reversed for 24 h; oscillation 170 rpm for 1 h; bake at 50°C for 10 min; swimming in 0°C water for 5 min; tail‐clamping for 1 min (Feng et al. 2023), as well as noise, dampness, restraint, or crowding, and so on. Of them, two different stressors were selected randomly each day and were different on two consecutive days. The CUMS mimics the role of stress in the etiology of anxiety–depression in human. CUMS‐induced depression might involve many factors, such as peripheral immune cells infiltration, microglial activation, and demyelination and synaptic deficited in hippocampus (Li et al. 2022); astrocyte disfunction (Yu et al. 2022; Li et al. 2023); sorting increased in prefrontal cortex and hippocampus (Chen et al. 2022); hippocampus undergo apoptosis (Yin et al. 2025); microglia reduced in the dentate gyrus of the hippocampus (Wang et al. 2025); dopamine neurons were inhibited in DRN, and so on (Wang et al. 2024). Other study has further showed the coexistence of brain and gut metabolic changes in CUMS‐induced depressive behavior in rats, which suggested a possible role of brain–gut axis in depression (Xu et al. 2022). A new study showed that CUMS‐induced depressive symptoms and gastric dysfunction, which related to the inhibition of GABAergic neurons in the bed nucleus of the stria terminalis (BNST) and excessive autophagy in the gastric tissues (Yuan et al. 2025). Another new study used CUMS + TC + IF for 28 days, also appearing depressive symptoms and gastric dysfunction, which may be related to hippocampal synaptic damage, and a decrease in brain–gut peptides and 5‐HT system. Moreover, the study selected sucrose preference rate, gastric emptying rate, and small intestinal propulsion rate as a criteria for establishing comorbidity model (Zhang et al. 2025). It can be seen that CUMS has certain advantages in the preparation of models for FD and emotional comorbidity.

**TABLE 2:** Emotional models with secondary GI dysfunction.

Methods	Animal species	Gastrointestinal function	Anxiety/Depressive behavior	Mechanism
Corticosterone (CORT) injection	KM mice	—	OFT: travel distance and time spent in center↓	Inflammatory activation of microglia, neuronal damage (Bai et al. 2022)
	C57BL/6J mice	Body weights↓	SPT: the sucrose preference↓ EPMT: open arm time spent↓	5‐HT↓ (Wu et al. 2023)
CORT + CRS	C57BL/6J mice	Body weights↓	EPMT: open arm time spent↓, OFT: time spent in center↓, FST: increased immobility	GABA,5‐HT↓ (Cai et al. 2022)
Chronic restraint stress (CRS)	Wistar rats	Body weight↓	OFT: time and entries into the center zone↓, EPMT: open arms time and entries↓	Synaptic connectivity of the CeA↓ (Moreno‐Martínez et al. 2022)
	ICR mice	—	OFT: fewer crossings and rearings, EPMT:time spent in the open arms↓	Lipid and amino acid metabolism (Yan et al. 2023)
	C57BL/6J male mice	—	FST: immobility↑, TST: immobility↑, OFT: time and entries into the center zone↓, EPMT: open arms time and entries↓	The dendritic spine density decreased (Tse et al. 2023)
CRS + IA	C57BL/6J mice	Gastric mucosal barrier disruption, goblet cells↓ gastric mucins↓, proinflammatory cytokines↑ microbiota disorder	OFT: The time in the center area↓, active exploration. TST: the immobility times↑	Gastrointestinal mucosal damage, disruption of gastrointestinal microbiota, disturbance of bile acid metabolism (Chen et al. 2024)
Chronic unpredictable mild stress (CUMS)	C57BL/6J mice	Intestinal propulsion rate↓, body weights↓, lamina propria cells of jejunum↑	TST: increased immobility, FST: increased immobility, SPT: the sucrose preference↓, OFT: reduced time and entries into the center zone	Focal demyelination in the medial prefrontal cortex (mPFC) (Li et al. 2022) attenuated astrocyte disfunction (Yu et al. 2022) The chemo genetic inhibition of DA neurons in the DRN (Wang et al. 2024) Aberrant synapse structure abnormality (Chen et al. 2022) Microglia↓ (Wang et al. 2025) CMS‐induced microglial activation and phagocytic activity in the mPFC (Li et al. 2023) BNSTGABA neurons are de‐suppressed (Yuan et al. 2025)
	SD rats	The diversity of the gut microbiota community↓, body weights↓.	SPT: sucrose preference↓, FST: immobility↑	Hippocampus apoptosis (Yin et al. 2025) altered metabolites in the hippocampus and jejunum (Xu et al. 2022)
CUMS + TC + IF	SD rats	Gastric emptying rate↓; small intestine propulsion rate↓	TST, FST: immobility time↑ SPT: sugar preference rat.↓	Abnormal myelin sheath structure and abnormal brain gut interaction (Zhang et al. 2025)

Abbreviations: 5‐HT, 5‐hydroxytryptamine; BNST GABA, bed nucleus of the stria terminalis GABAergic; CeA, central amygdaloid nucleus; DA, dopamine; DRN, dorsal raphe nucleus; EPMT, elevated plus maze test; FST, forced swimming test; GABA, γ‐aminobutyric acid; IA, iodoacetamide; KM, Kunming; mPFC, medial prefrontal cortex; OFT, open field test; SD, Sprague‐Dawley; SPT, sucrose preference test; TST, tail suspension test.

## 6 Possible Gastrointestinal‐Emotional Comorbidity Models (Shown as Table 3)

Although the independent FD and depression models have been clarified, few validating comorbidity models mimic both gastrointestinal dysfunction and emotional disturbances, which limits mechanism‐based therapy discovery. This study is the first comprehensive comparison of FD and anxiety–depression modeling protocols to propose effective protocols of comorbidity models.

First, we find that IA gavage is a classical chemical method for establishing FD model with gastric hypersensitivity; TC has also been used frequently for FD model, which simulates the psychological stress; physical stress such as “ice water gavage” or “IF” aims to simulate the unhealthy dietary habits of modern humans, partially participating in the pathological process of FD. It is often combined with other methods. Therefore, we propose that IA + TC + IF or TC + IF + ice water gavage can be used to prepare comorbidity models in the future. The experimental protocols can better simulate the clinical pathogenesis. However, the exact experimental process, especially the amount and duration of stimulation, needs to be further explored. It is recommended to dynamically observe the process of the model with different stimulation amounts and different time nodes to optimize experiment parameters.

Second, we discover that MS can induce offspring mice to exhibit gastric hypersensitivity and anxiety‐like behavior, and maternal mice appear higher depressive and anxiety‐like behaviors. So, MS may potentially become one of the effective models for studying comorbidities in the future. However, when researchers choose MS to establish comorbidity model, they should take into account the long duration and the issue of hormone interference.

Third, we reveal that both CRS and CUMS are classical stress models with anxiety and depressive‐like behavior. The stress can induce emotional disorders maybe by disturbing the emotion‐related centers such as CeA, hippocampus mPFC, PVT, NAc, LHb, BLA, DRN, or BNST. CUMS simulates the multifactorial stresses in the etiology of depression in humans, but CUMS includes many different stressors, and different protocols select stressors that are also not the same. As a result, there is currently no unified, reproducible, and stable induction model. CRS mimics a single stress, and the advantage of the protocol is stable and easy to be replicated. Moreover, CRS combined with IA gavage or IF can induce GMD; CUMS + TC + IF can induce depressive symptoms and gastric dysfunction. So, we suggest CRS/CUMS (protocol of classic anxiety–depression model) combine with IA/IF/TC (protocol of classic gastric dysfunction model) to establish gastrointestinal ‐emotional comorbidity model through brain–gut cross talk.

**TABLE 3:** Possible gastrointestinal‐emotional comorbidity models.

Model type	Core method	Anxiety/Depression indicators	Gastrointestinal indicators	Advantages	Limitations	Recommendations
IA + TC + IF	Two‐stage multifactors approach, first stage: IA gavage in neonatal rats/mice for 6 days; second stage: TC combined with IF at 7‐week‐old rats/mice for 14/21 days	General behavior: activity level, irritable anxious; infighting exhausted, sluggish, uncomfortable Anxiety/Depressive behavior: OFT/SPT/TST BDNF/5‐HT/DA levels	Gastric motility/gastric emptying intestinal propulsion rate Intestinal permeability Visceral hypersensitivity Gut microbiota Gastrointestinal hormone (MTL/GAS/VIP/NO/CCK‐8) Gastric acid and pepsin	IA gavage and TC are classical chemical/psychological stress. IF simulate irregular food. The three‐factors combination can better to simulate clinical pathogenesis	The modeling cycle is long (newborn IA gavage + adult TC + IF) The amount and duration of stimulation of the comorbidity model are not clarify	To dynamic observe the indicators of the model with different stimulation amounts and different time nodes to optimize experiment parameters
TC + IF + 0°C water gavage	TC + IF + 0°C water gavage for 14/21/28 days	Anxiety/Depressive behavior: OFT/EMP/SPT/TST/FST Emotional center‐PVN activity Serum CRF, BDNF, 5‐HT levels	Gastric residual rate/gastric emptying Intestinal propulsion rate Gastrointestinal inflammatory response Gut microbiota Gastrointestinal hormone	TC simulates psychological stress, IF and 0°C water gavage simulate unhealthy dietary habits of modern humans, which is according to the causes of comorbidity	The standard experimental tests of anxiety and depression are not sufficient The amount and duration of stimulation of the comorbidity model are still to explore	Improving the indicators of comorbidity model Multi‐time node tests are used to define the time when the model is successful
MS	Newborn rats/mice are separated from mother for 2 h a day from postnatal Days 1–10. At 7–8 week of age, observing gastric function and mood changes	Anxiety/Depressive behavior: OFT/SPT/TST Emotional center‐mPFC/hippocampal activity	Gastric hypersensitivity Gastroduodenal microinflammation	Both maternal mice and their offspring can be simultaneously observed for comorbidity states Sex factors can be included in the study	The modeling cycle is long (from newborn to adulthood) The model may be influenced by hormone	It is suitable for the study of anxiety and depression in female
CRS + IA/TC/IF	CRS for 14/21 days +IA/TC/IF (same as above)	Anxiety‐like behavior: OFT, EPM Depressive‐like behavior: SPT/TST/FST GABA/Glu, 5‐HT, and NE levels Emotional center‐CeA//PVT/LHb/mPFC/hippocampal activity	Gastric mucosal barrier Gastric motility Proinflammatory cytokines Gut microbiota Food intake	CRS exhibits high stability and is easily reproducible	Currently, CRS is mainly used for establishing anxiety/depression model. CRS + IA/TC/IF for comorbidity model needing more experimental exploration in future	To study CRS combined with one or more IA/TC/IF can better establish a comorbidity model by animal experiments
CUMS + TC/IF/IA	CUMS for 28 days +IA/TC/IF (same as above)	Depressive‐like behavior: SPT/TST/FST Brain–gut peptides, DA/5‐HT neurotransmitters	Gastric emptying rate and small intestinal propulsion rate Body weights Gastric motility Food intake	CUMS simulates the multifactorial stresses in the etiology of depression in humans	CUMS include many different stressors, and different protocols select stressors also not the same, thus inducing model with repeatability and stability not good enough	Standardize stressors of CUMS

Abbreviations: 5‐HT, 5‐hydroxytryptamine; BDNF, brain‐derived neurotrophic factor; CCK‐8, cell counting kit‐8; CeA, carcinoembryonic antigen; DA, dopamine; FST, forced swim test; GABA, gamma‐aminobutyric acid; GAS, gastrin; Glu, glucose; LHb, lateral habenular nucleus; mPFC, medial prefrontal cortex; MTL, motilin; NE, norepinephrine; NO, nitric oxide; OFT, ail suspension test; PVT, production verification test; SPT, sucrose preference test; TST, tail suspension test; VIP, vasoactive intestinal polypeptide.

## 7 Conclusion

The exploration of comorbidity models between FD and anxiety–depression has become a significant direction in preclinical research. The two diseases do not exist independently, but instead form a complex comorbidity system characterized by bidirectional regulation. This phenomenon involves the interaction of multiple biological, psychological, and social factors. However, most current animal models still struggle to fully replicate the pathological features observed in clinical practice causally intertwined and interconnected, which has significantly limited researchers’ ability to gain a deeper understanding of its mechanisms and develop therapeutic strategies.

The review systematically compares and analyzes FD and depression models, aiming to provide suggestions for establishing comorbidity models. The results show that the gastrointestinal‐emotional comorbidity model protocol has initially formed, but there are still shortcomings, mainly appearing in three aspects: First, there is no uniform standard for modeling time. According to the statistics, FD models usually last 7 or 14 days, whereas depression models often sustain 21 or 28 days. It is recommended to systematically evaluate the behavioral indicators at different time nodes of 7, 14, 21, and 28 days to dynamically evaluate the best duration of comorbidity models.

Second, the evaluation indicators are not comprehensive enough. It is recommended to record gastric emptying rate, small intestine propulsion rate, and visceral sensitivity with gastrointestinal dysfunction; sucrose preference, FS, and open field tests with depressive‐like behavior. However, it also includes multidimensional parameters such as the microbiota, neuroinflammation, metabolomics, brain–gut peptide, and neurotransmitters based on different comorbidity models characteristics. In the future, we can also validate comorbidity models with pharmacological interventions (e.g., antidepressants, probiotics, and prokinetics) and explore noninvasive physiological monitoring (e.g., telemetry and imaging) for real‐time gut–brain dynamics. Therefore, we aim to develop a comprehensive and standardized evaluation system for comorbidity models.

Finally, there are differences in experimental animal selection. FD models predominantly utilize SD rats, whereas anxiety and depression models tend to select C57 mice. It is recommended to choose the appropriate animal species according to the research purpose. In addition, there is an obvious gender imbalance problem in the current research. Considering the higher prevalence of FD and mood disorders in females, future modeling efforts should prioritize inclusion of female animals to better replicate human disease patterns, thus improving the clinical translation value of the research.

In conclusion, the review identifies critical overlaps between models of FD and anxiety–depression, highlighting stress as a shared etiological driver mediated via gut–brain–immune mechanisms. The establishment of standardized, multidimensional comorbidity models that incorporate microbial, neuroimmune, and behavioral parameters will be essential to elucidate pathophysiological mechanisms and advance the development of new therapies.

## Author Contributions

Hao Wang designed and revised the manuscript. Yan‐hui Wang and Xiang‐rui Kong wrote the manuscript. Xin‐zhu Ye collected literature and created tables. Su Pu, Xin‐xin Ren, Qing‐yang Huang, and Ya‐qing Lu collected information.

## Funding

The study was supported by the Excellent Youth Project of Anhui Universities (2022AH030065); Open projects of Anhui Province Key Laboratory of Meridian Viscera Correlation ship (2024AHMVC04); Anhui Provincial Natural Science Foundation (2408085MH223); Key teaching and research projects of Anhui University of Chinese Medicine (2023xjjy_zd025).

## Conflicts of Interest

The authors declare no conflicts of interest.

## Data Availability

Data sharing is not applicable to this article as no datasets were generated or analyzed during the current study.

## References

[brb371135-bib-0001] Aburto, M. R. , and J. F. Cryan . 2024. “Gastrointestinal and Brain Barriers: Unlocking Gates of Communication Across the Microbiota‐Gut‐Brain Axis.” Nature Reviews Gastroenterology & Hepatology 21, no. 4: 222–247. 10.1038/s41575-023-00890-0. 38355758

[brb371135-bib-0002] Accarie, A. , J. Toth , L. Wauters , R. Farré , J. Tack , and T. Vanuytsel . 2022. “Estrogens Play a Critical Role in Stress‐Related Gastrointestinal Dysfunction in a Spontaneous Model of Disorders of Gut‐Brain Interaction.” Cells 11, no. 7: 1214. 10.3390/cells11071214. 35406778 PMC8997409

[brb371135-bib-0003] Accarie, A. , and T. Vanuytsel . 2020. “Animal Models for Functional Gastrointestinal Disorders.” Front Psychiatry 11: 509681. 10.3389/fpsyt.2020.509681. 33262709 PMC7685985

[brb371135-bib-0004] Agirman, G. , K. B. Yu , and E. Y. Hsiao . 2021. “Signaling Inflammation Across the Gut‐Brain Axis.” Science 374, no. 6571: 1087–1092. 10.1126/science.abi6087. 34822299

[brb371135-bib-0005] Akers, K. G. , M. Arruda‐Carvalho , S. A. Josselyn , and P. W. Frankland . 2012. “Ontogeny of Contextual Fear Memory Formation, Specificity, and Persistence in Mice.” Learning & Memory (Cold Spring Harbor, New York) 19, no. 12: 598–604. 10.1101/lm.027581.112. 23161449

[brb371135-bib-0006] Bai, G. , Y. Qiao , P.‐C. Lo , et al. 2022. “Anti‐Depressive Effects of Jiao‐Tai‐Wan on CORT‐Induced Depression in Mice by Inhibiting Inflammation and Microglia Activation.” Journal of Ethnopharmacology 283: 114717. 10.1016/j.jep.2021.114717. 34627986

[brb371135-bib-0007] Bai, M. , L. Zhao , M. Liu , et al. 2024. “Deciphering the Function of Xiangsha‐Liujunzi‐Tang in Enhancing Duodenal Mucosal Barrier by Inhibiting MC/Tryptase/PAR‐2 Signaling Pathway in Functional Dyspepsia Rats.” Journal of Ethnopharmacology 319, no. pt 1: 116715. 10.1016/j.jep.2023.116715. 37308030

[brb371135-bib-0008] Bai, Y. , M. Zheng , R. Fu , et al. 2023. “Effect of Massa Medicata Fermentata on the Intestinal Flora of Rats With Functional Dyspepsia.” Microbial Pathogenesis 174: 105927. 10.1016/j.micpath.2022.105927. 36529285

[brb371135-bib-0009] Barilla, R. M. , C. Berard , L. Sun , et al. 2025. “Type 2 Cytokines Act on Enteric Sensory Neurons to Regulate Neuropeptide‐Driven Host Defense.” Science 389, no. 6757: 260–267. 10.1126/science.adn9850. 40403128 PMC12632183

[brb371135-bib-0010] Cai, L. , Q. Tao , W. Li , X. Zhu , and C. Cui . 2022. “The Anti‐Anxiety/Depression Effect of a Combined Complex of Casein Hydrolysate and Gamma‐Aminobutyric Acid on C57BL/6 Mice.” Frontiers in Nutrition 9: 971853. 10.3389/fnut.2022.971853. 36245498 PMC9554304

[brb371135-bib-0011] Chen, M. , Y. Li , L. Li , et al. 2024. “Qi‐Zhi‐Wei‐Tong Granules Alleviates Chronic Non‐Atrophic Gastritis in Mice by Altering the Gut Microbiota and Bile Acid Metabolism.” Journal of Ethnopharmacology 319, no. pt 3: 117304. 10.1016/j.jep.2023.117304. 37838294

[brb371135-bib-0012] Chen, S.‐J. , C.‐C. Gao , Q.‐Y. Lv , M.‐Q. Zhao , X.‐Y. Qin , and H. Liao . 2022. “Sortilin Deletion in the Prefrontal Cortex and Hippocampus Ameliorates Depressive‐Like Behaviors in Mice via Regulating ASM/Ceramide Signaling.” Acta Pharmacologica Sinica 43, no. 8: 1940–1954. 10.1038/s41401-021-00823-0. 34931016 PMC9343424

[brb371135-bib-0013] Chen, S.‐H. , L.‐J. Zhu , Y.‐H. Zhi , et al. 2023. “Pitongshu Alleviates the Adverse Symptoms in Rats With Functional Dyspepsia Through Regulating Visceral Hypersensitivity Caused by 5‐HT Overexpression.” Combinatorial Chemistry & High Throughput Screening 26, no. 7: 1424–1436. 10.2174/1386207325666220827152654. 36043772 PMC10234081

[brb371135-bib-0014] Chen, Y. , Y. Zhao , R. Y. Tan , et al. 2021. “The Influence of Stomach Back‐Shu and Front‐Mu Points on Insular Functional Connectivity in Functional Dyspepsia Rat Models.” Evidence‐Based Complementary and Alternative Medicine 2021: 2771094. 34621320 10.1155/2021/2771094 PMC8490795

[brb371135-bib-0015] Cordner, Z. A. , Q. Li , L. Liu , et al. 2021. “Vagal Gut‐Brain Signaling Mediates Amygdaloid Plasticity, Affect, and Pain in a Functional Dyspepsia Model.” JCI Insight 6, no. 6: e144046. 10.1172/jci.insight.144046. 33591956 PMC8026195

[brb371135-bib-0016] Deng, Y. , M. Zhou , J. Wang , et al. 2021. “Involvement of the Microbiota‐Gut‐Brain Axis in Chronic Restraint Stress: Disturbances of the Kynurenine Metabolic Pathway in Both the Gut and Brain.” Gut Microbes 13, no. 1: 1–16. 10.1080/19490976.2020.1869501. PMC7872056 33535879

[brb371135-bib-0017] Dong, J. Z. , P. J. Rong , T. M. Ma , D. Wang , X. T. Wang , and Y. Qiao . 2022. “[Influence of Electroacupuncture of “Zusanli” (ST36) on Mast Cells/TRPV1 Signaling Pathway in Visceral Hypersensitivity Rats With Functional Dyspepsia].” Zhen Ci Yan Jiu 47, no. 7: 592–597. 35880275 10.13702/j.1000-0607.20210937

[brb371135-bib-0018] Dong, W.‐Y. , X. Zhu , H.‐D. Tang , et al. 2023. “Brain Regulation of Gastric Dysfunction Induced by Stress.” Nature Metabolism 5, no. 9: 1494–1505. 10.1038/s42255-023-00866-z. 37592008

[brb371135-bib-0019] Duan, S. , N. Imamura , T. Kondo , et al. 2022. “Yokukansan Suppresses Gastric Hypersensitivity and Eosinophil‐Associated Microinflammation in Rats With Functional Dyspepsia.” Journal of Neurogastroenterology and Motility 28, no. 2: 255–264. 10.5056/jnm21204. 35362452 PMC8978130

[brb371135-bib-0020] Duan, S. , T. Kondo , H. Miwa , et al. 2021. “Eosinophil‐Associated Microinflammation in the Gastroduodenal Tract Contributes to Gastric Hypersensitivity in a Rat Model of Early‐Life Adversity.” American Journal of Physiology Gastrointestinal and Liver Physiology 320, no. 2: G206–G216. 10.1152/ajpgi.00313.2020. 33174456

[brb371135-bib-0021] Esterita, T. , S. Dewi , F. G. Suryatenggara , and G. Glenardi . 2021. “Association of Functional Dyspepsia With Depression and Anxiety: A Systematic Review.” Journal of Gastrointestinal and Liver Diseases 30, no. 2: 259–266. 10.15403/jgld-3325. 33951117

[brb371135-bib-0022] Feng, S. , C. Meng , Y. Liu , et al. 2023. “ *Bacillus licheniformis* Prevents and Reduces Anxiety‐Like and Depression‐Like Behaviours.” Applied Microbiology and Biotechnology 107, no. 13: 4355–4368. 10.1007/s00253-023-12580-7. 37209162

[brb371135-bib-0023] Guo, Y. , and P. Gharibani . 2024. “Analgesic Effects of Vagus Nerve Stimulation on Visceral Hypersensitivity: A Direct Comparison Between Invasive and Noninvasive Methods in Rats.” Neuromodulation 27, no. 2: 284–294. 10.1016/j.neurom.2023.04.001. 37191611

[brb371135-bib-0024] Han, J. , W. Wei , H. C. Wang , et al. 2022. “[Transcutaneous Auricular Vagus Nerve Stimulation Promotes Gastric Motility by Up‐Rgulating alpha7nAChR and Suppressing NF‐kappaB p65 Expression in Duodenum in Rats With Functional Dyspepsia].” Zhen Ci Yan Jiu 47, no. 6: 517–524. 35764519 10.13702/j.1000-0607.20220111

[brb371135-bib-0025] Han, Y.‐L. , X.‐M. Peng , H.‐X. Zhang , S. Chen , and L.‐Y. Zhang . 2021. “Electroacupuncture Regulates TRPV1 Through PAR2/PKC Pathway to Alleviate Visceral Hypersensitivity in FD Rats.” Evidence‐Based Complementary and Alternative Medicine 2021: 1–10. 10.1155/2021/1975228. PMC8648456 34880917

[brb371135-bib-0026] He, Y. , Y. Ren , X. Chen , et al. 2025. “Neural and Molecular Investigation Into the Paraventricular Thalamus for Chronic Restraint Stress Induced Depressive‐Like Behaviors.” Journal of Advanced Research 75: 521–537. 10.1016/j.jare.2024.10.025.39447640

[brb371135-bib-0027] Hou, L.‐W. , J.‐L. Fang , J.‐L. Zhang , et al. 2022. “Auricular Vagus Nerve Stimulation Ameliorates Functional Dyspepsia With Depressive‐Like Behavior and Inhibits the Hypothalamus‐Pituitary‐Adrenal Axis in a Rat Model.” Digestive Diseases and Sciences 67, no. 10: 4719–4731. 10.1007/s10620-021-07332-4. 35064375

[brb371135-bib-0028] Kraimi, N. , T. Ross , J. Pujo , and G. De Palma . 2024. “The Gut Microbiome in Disorders of Gut‐Brain Interaction.” Gut Microbes 16, no. 1: 2360233. 10.1080/19490976.2024.2360233. 38949979 PMC11218806

[brb371135-bib-0029] Li, C. , B. Liu , J. Xu , et al. 2023. “Phloretin Decreases Microglia‐Mediated Synaptic Engulfment to Prevent Chronic Mild Stress‐Induced Depression‐Like Behaviors in the mPFC.” Theranostics 13, no. 3: 955–972. 10.7150/thno.76553. 36793870 PMC9925308

[brb371135-bib-0030] Li, Y. , P. Su , Y. Chen , et al. 2022. “The Eph Receptor A4 Plays a Role in Demyelination and Depression‐Related Behavior.” Journal of Clinical Investigation 132, no. 8: e152187. 10.1172/JCI152187. 35271507 PMC9012277

[brb371135-bib-0031] Liu, T. , I. M. Asif , L. Liu , M. Zhang , B. Li , and L. Wang . 2024. “Laminarin Ameliorates Iodoacetamide‐Induced Functional Dyspepsia via Modulation of 5‐HT(3) Receptors and the Gut Microbiota.” International Journal of Biological Macromolecules 268, no. pt. 1: 131640. 10.1016/j.ijbiomac.2024.131640. 38636750

[brb371135-bib-0032] Liu, W. W. , N. Reicher , E. Alway , et al. 2025. “A Gut Sense for a Microbial Pattern Regulates Feeding.” Nature 645, no. 8081: 729–736. 10.1038/s41586-025-09301-7. 40702192 PMC12443592

[brb371135-bib-0033] Margolis, K. G. , J. F. Cryan , and E. A. Mayer . 2021. “The Microbiota‐Gut‐Brain Axis: From Motility to Mood.” Gastroenterology 160, no. 5: 1486–1501. 10.1053/j.gastro.2020.10.066. 33493503 PMC8634751

[brb371135-bib-0034] Moreno‐Martínez, S. , H. Tendilla‐Beltrán , V. Sandoval , G. Flores , and J. A. Terrón . 2022. “Chronic Restraint Stress Induces Anxiety‐Like Behavior and Remodeling of Dendritic Spines in the Central Nucleus of the Amygdala.” Behavioural Brain Research 416: 113523. 10.1016/j.bbr.2021.113523. 34390801

[brb371135-bib-0035] Noori, M. , A. Hasbi , M. Sivasubramanian , M. Milenkovic , and S. R. George . 2020. “Maternal Separation Model of Postpartum Depression: Potential Role for Nucleus Accumbens Dopamine D1–D2 Receptor Heteromer.” Neurochemical Research 45, no. 12: 2978–2990. 10.1007/s11064-020-03145-5. 33057844

[brb371135-bib-0036] Ouyang, X. , S. Li , J. Zhou , and J. D. Chen . 2020. “Electroacupuncture Ameliorates Gastric Hypersensitivity via Adrenergic Pathway in a Rat Model of Functional Dyspepsia.” Neuromodulation 23, no. 8: 1137–1143. 10.1111/ner.13154. 32282996

[brb371135-bib-0037] Pan, J. , J. Wu , S. Zhang , et al. 2024. “Targeted Metabolomics Revealed the Mechanisms Underlying the Role of Liansu Capsule in Ameliorating Functional Dyspepsia.” Journal of Ethnopharmacology 321: 117568. 10.1016/j.jep.2023.117568. 38092317

[brb371135-bib-0038] Person, H. , and L. Keefer . 2021. “Psychological Comorbidity in Gastrointestinal Diseases: Update on the Brain‐Gut‐Microbiome Axis.” Progress in Neuro‐Psychopharmacology & Biological Psychiatry 107: 110209. 10.1016/j.pnpbp.2020.110209. 33326819 PMC8382262

[brb371135-bib-0039] Qiao, Y. , H. Chen , J. Guo , et al. 2025. “A Study of Sex Differences in the Biological Pathways of Stress Regulation in Mice.” CNS Neuroscience & Therapeutics 31, no. 5: e70433. 10.1111/cns.70433. 40365748 PMC12076126

[brb371135-bib-0040] Ruan, X. , J. Chen , Y. Sun , et al. 2023. “Depression and 24 Gastrointestinal Diseases: A Mendelian Randomization Study.” Translational Psychiatry 13, no. 1: 146. 10.1038/s41398-023-02459-6. 37142593 PMC10160129

[brb371135-bib-0041] Song, J. , T. Wang , X. Zhang , B. Li , C. Zhu , and S. Zhang . 2020. “Upregulation of Gastric Norepinephrine With Beta‐Adrenoceptors and Gastric Dysmotility in a Rat Model of Functional Dyspepsia.” Physiological Research 69, no. 1: 135–143. 10.33549/physiolres.934169. 31852208 PMC8565965

[brb371135-bib-0042] Sun, R. , Z. He , P. Ma , et al. 2021. “The Participation of Basolateral Amygdala in the Efficacy of Acupuncture With Deqi Treating for Functional Dyspepsia.” Brain Imaging and Behavior 15, no. 1: 216–230. 10.1007/s11682-019-00249-7. 32125619

[brb371135-bib-0043] Tang, L. , Y. Zeng , L. Li , et al. 2020. “Electroacupuncture Upregulated Ghrelin in Rats With Functional Dyspepsia via AMPK/TSC2/Rheb‐Mediated mTOR Inhibition.” Digestive Diseases and Sciences 65, no. 6: 1689–1699. 10.1007/s10620-019-05960-5. 31863340 PMC7225202

[brb371135-bib-0044] Tse, W. S. , B. Pochwat , B. Szewczyk , et al. 2023. “Restorative Effect of NitroSynapsin on Synaptic Plasticity in an Animal Model of Depression.” Neuropharmacology 241: 109729. 10.1016/j.neuropharm.2023.109729. 37797736

[brb371135-bib-0045] Tu, Y. , X. Luo , D. Liu , et al. 2022. “Extracts of Poria Cocos Improve Functional Dyspepsia via Regulating Brain‐Gut Peptides, Immunity and Repairing of Gastrointestinal Mucosa.” Phytomedicine 95: 153875. 10.1016/j.phymed.2021.153875. 34911003

[brb371135-bib-0046] Wang, D. , J. Zhang , D. Yang , et al. 2023. “Electroacupuncture Restores Intestinal Mucosal Barrier Through TLR4/NF‐kappaB p65 Pathway in Functional Dyspepsia‐Like Rats.” Anatomical Record (Hoboken) 306, no. 12: 2927–2938. 10.1002/ar.24800. 34713984

[brb371135-bib-0047] Wang, H. , W.‐J. Liu , M.‐J. Hu , M.‐T. Zhang , and G.‐M. Shen . 2020. “Acupuncture at Gastric Back‐Shu and Front‐Mu Acupoints Enhances Gastric Motility via the Inhibition of the Glutamatergic System in the Hippocampus.” Evidence‐Based Complementary and Alternative Medicine 2020: 3524641. 10.1155/2020/3524641. 32215036 PMC7085822

[brb371135-bib-0048] Wang, H. , W.‐J. Liu , X.‐Y. Wang , et al. 2023. “A Central Amygdala Input to the Dorsal Vagal Complex Controls Gastric Motility in Mice Under Restraint Stress.” Frontiers in Physiology 14: 1074979. 10.3389/fphys.2023.1074979. 36875016 PMC9975572

[brb371135-bib-0049] Wang, H. , J. Peng , H. Zhu , et al. 2025. “Microglia Stimulation Produces Antidepressant‐Like Effects in a Mouse Depression Model Induced by Adolescent Chronic Unpredictable Stress.” Physiology & Behavior 291: 114782. 10.1016/j.physbeh.2024.114782. 39672484

[brb371135-bib-0050] Wang, H. , Y.‐Z. Tan , R.‐H. Mu , et al. 2021. “Takeda G Protein‐Coupled Receptor 5 Modulates Depression‐Like Behaviors via Hippocampal CA3 Pyramidal Neurons Afferent to Dorsolateral Septum.” Biological Psychiatry 89, no. 11: 1084–1095. 10.1016/j.biopsych.2020.11.018. 33536132

[brb371135-bib-0051] Wang, W. , D. Wang , D. Zhao , et al. 2024. “Dorsal Raphe Dopaminergic Neurons Target CaMKII(+) Neurons in Dorsal Bed Nucleus of the Stria Terminalis for Mediating Depression‐Related Behaviors.” Translational Psychiatry 14, no. 1: 408. 10.1038/s41398-024-03093-6. 39358336 PMC11447211

[brb371135-bib-0052] Wang, X. , L. Jiang , W. Ma , et al. 2022. “Maternal Separation Affects Anxiety‐Like Behavior Beginning in Adolescence and Continuing Through Adulthood and Related to Dnmt3a Expression.” Journal of Neurophysiology 128, no. 3: 611–618. 10.1152/jn.00247.2022. 35946792

[brb371135-bib-0053] Wang, X. , X. Liu , Y. Wang , et al. 2024. “Chaihu Shugan Powder Inhibits Interstitial Cells of Cajal Mitophagy Through USP30 in the Treatment of Functional Dyspepsia.” Journal of Ethnopharmacology 323: 117695. 10.1016/j.jep.2023.117695. 38163556

[brb371135-bib-0054] White, Z. , I. Cabrera , L. Mei , et al. 2025. “Gut Inflammation Promotes Microbiota‐Specific CD4 T Cell‐Mediated Neuroinflammation.” Nature 643, no. 8071: 509–518. 10.1038/s41586-025-09120-w. 40533562

[brb371135-bib-0055] Wu, C. , J. He , Y. Zhu , et al. 2023. “Ultrasound Neuromodulation Ameliorates Chronic Corticosterone‐Induced Depression‐ and Anxiety‐Like Behaviors in Mice.” Journal of Neural Engineering 20, no. 3: 036037. 10.1088/1741-2552/acdea9. 37321207

[brb371135-bib-0056] Wu, L. U. , Y. Lai , Y. Wang , L. Tan , L. Wen , and H. Yang . 2020. “Maillard Reaction Products of Stir Fried Hordei Fructus Germinatus Are Important for Its Efficacy in Treating Functional Dyspepsia.” Journal of Medicinal Food 23, no. 4: 420–431. 10.1089/jmf.2019.4430. 31971858

[brb371135-bib-0057] Wu, Y.‐Y. , Z.‐S. Zhong , Z.‐H. Ye , et al. 2021. “D‐Galacturonic Acid Ameliorates the Intestinal Mucosal Permeability and Inflammation of Functional Dyspepsia in Rats.” Annals of Palliative Medicine 10, no. 1: 538–548. 10.21037/apm-20-2420. 33440961

[brb371135-bib-0058] Wu, Z. , X. Lu , S. Zhang , and C. Zhu . 2020. “Sini‐San Regulates the NO‐cGMP‐PKG Pathway in the Spinal Dorsal Horn in a Modified Rat Model of Functional Dyspepsia.” Evidence‐Based Complementary and Alternative Medicine 2020: 3575231. 10.1155/2020/3575231. 32328126 PMC7150674

[brb371135-bib-0059] Xiao, H.‐L. , Y.‐J. Xiao , Q. Wang , M.‐L. Chen , and A.‐L. Jiang . 2021. “Moxibustion Regulates Gastrointestinal Motility via HCN1 in Functional Dyspepsia Rats.” Medical Science Monitor 27: e932885. 10.12659/MSM.932885. 34845181 PMC8642983

[brb371135-bib-0060] Xiao, Y. , J. Y. Zhou , H. Z. Yin , et al. 2023. “Effect of Electroacupuncture at “Neiguan” (PC 6) and “Zusanli” (ST 36) on Gastrointestinal Hormone in the Antral Tissue of Rats With Functional Dyspepsia.” Zhongguo Zhen Jiu 43, no. 12: 1435–1440. 38092545 10.13703/j.0255-2930.20230809-0007

[brb371135-bib-0061] Xiong, R. , Y. Hu , M. Wang , et al. 2025. “Peripheral CD4(+) T Cells Mediate the Destructive Effects of Maternal Separation on Prefrontal Myelination and Cognitive Functions.” PNAS 122, no. 16: e2412995122. 10.1073/pnas.2412995122. 40238461 PMC12037062

[brb371135-bib-0062] Xu, Q. , M. Jiang , S. Gu , et al. 2022. “Metabolomics Changes in Brain‐Gut Axis After Unpredictable Chronic Mild Stress.” Psychopharmacology 239, no. 3: 729–743. 10.1007/s00213-021-05958-w. 35133451 PMC8891102

[brb371135-bib-0063] Yan, L. , F. Yang , Y. Wang , et al. 2024. “Stress Increases Hepatic Release of Lipocalin 2 Which Contributes to Anxiety‐Like Behavior in Mice.” Nature Communications 15, no. 1: 3034. 10.1038/s41467-024-47266-9. PMC11001612 38589429

[brb371135-bib-0064] Yan, Y. , J. Li , Y. Zhang , et al. 2023. “Screening the Effective Components of Suanzaoren Decoction on the Treatment of Chronic Restraint Stress Induced Anxiety‐Like Mice by Integrated Chinmedomics and Network Pharmacology.” Phytomedicine 115: 154853. 10.1016/j.phymed.2023.154853. 37156059

[brb371135-bib-0065] Yang, L. , C. Guo , Z. Zheng , et al. 2025. “Stress Dynamically Modulates Neuronal Autophagy to Gate Depression Onset.” Nature 641, no. 8062: 427–437. 10.1038/s41586-025-08807-4. 40205038 PMC12058529

[brb371135-bib-0066] Ye, X. , M. Zhang , N. Zhang , H. Wei , and B. Wang . 2024. “Gut‐Brain Axis Interacts With Immunomodulation in Inflammatory Bowel Disease.” Biochemical Pharmacology 219: 115949. 10.1016/j.bcp.2023.115949. 38036192

[brb371135-bib-0067] Yin, L. , H. Wang , H. Xu , H. Lu , J. Lv , and C. Lu . 2025. “Asperuloside Suppresses the Progression of Depression Through O‐GlcNAcylation of IκBα and Regulating NFκB Signaling.” Journal of Pharmacological Sciences 157, no. 3: 179–188. 10.1016/j.jphs.2025.01.010. 39929592

[brb371135-bib-0068] Yu, X. , Y. Bai , B. Han , et al. 2022. “Extracellular Vesicle‐Mediated Delivery of circDYM Alleviates CUS‐Induced Depressive‐Like Behaviours.” Journal of Extracellular Vesicles 11, no. 1: e12185. 10.1002/jev2.12185. 35029057 PMC8758833

[brb371135-bib-0069] Yuan, Y. , J. Xu , S. Zhu , et al. 2025. “BNST(GABA) Neurons Regulate Autophagy to Alleviate Depression With Gastric Dysfunction Symptoms.” Brain Research Bulletin 226: 111360. 10.1016/j.brainresbull.2025.111360. 40294829

[brb371135-bib-0070] Żakowski, W. 2020. “Animal Use in Neurobiological Research.” Neuroscience 433: 1–10. 10.1016/j.neuroscience.2020.02.049. 32156550

[brb371135-bib-0071] Zeng, J. , M. Li , Z. You , et al. 2025. “Acupuncture Improves Depression‐Like Behaviors in Rats Through Gut Microbiota and TLR4/MyD88/NF‐kappaB Pathway Modulation.” Brain Research Bulletin 232: 111590. 10.1016/j.brainresbull.2025.111590. 41130501

[brb371135-bib-0072] Zeng, Y. , L. Zhou , Y. Wan , et al. 2024. “Effects of Saikosaponin D on Apoptosis, Autophagy, and Morphological Structure of Intestinal Cells of Cajal With Functional Dyspepsia.” Combinatorial Chemistry & High Throughput Screening 27, no. 10: 1513–1522. 10.2174/0113862073262404231004053116. 37818570 PMC11340291

[brb371135-bib-0073] Zhang, C. , G. Q. Zhu , J. J. Wang , X. J. Li , M. Li , and X. C. Wang . 2025. “[Study on the Mechanism of Acupuncture Underlying Improvement of Functional Dyspepsia With Depression‐Like Behavior in Rats].” Zhen Ci Yan Jiu 50, no. 1: 76–83. 39961761 10.13702/j.1000-0607.20230948

[brb371135-bib-0074] Zhang, J. , X. Wang , F. Wang , and X. Tang . 2022. “Xiangsha Liujunzi Decoction Improves Gastrointestinal Motility in Functional Dyspepsia With Spleen Deficiency Syndrome by Restoring Mitochondrial Quality Control Homeostasis.” Phytomedicine 105: 154374. 10.1016/j.phymed.2022.154374. 35963194

[brb371135-bib-0075] Zhang, M.‐T. , Y.‐F. Liang , Q. Dai , et al. 2025. “A Spinal Neural Circuit for Electroacupuncture That Regulates Gastric Functional Disorders.” Journal of Integrative Medicine 23, no. 1: 56–65. 10.1016/j.joim.2024.11.005. 39694773

[brb371135-bib-0076] Zhang, N. , S. Zhao , Y. Ma , et al. 2024. “Hyperexcitation of ovBNST CRF Neurons During Stress Contributes to Female‐Biased Expression of Anxiety‐Like Avoidance Behaviors.” Science Advances 10, no. 19: eadk7636. 10.1126/sciadv.adk7636. 38728397 PMC11086623

[brb371135-bib-0077] Zhang, S. , Z. Xu , X. Cao , et al. 2020. “Shenling Baizhu San Improves Functional Dyspepsia in Rats as Revealed by (1)H‐NMR Based Metabolomics.” Analytical Methods 12, no. 18: 2363–2375. 10.1039/D0AY00580K. 32930262

[brb371135-bib-0078] Zhou, Y. , M. Yan , R. Pan , et al. 2021. “Radix Polygalae Extract Exerts Antidepressant Effects in Behavioral Despair Mice and Chronic Restraint Stress‐Induced Rats Probably by Promoting Autophagy and Inhibiting Neuroinflammation.” Journal of Ethnopharmacology 265: 113317. 10.1016/j.jep.2020.113317. 32861821

[brb371135-bib-0079] Zhu, C. , L. Zhao , J. Zhao , and S. Zhang . 2020. “Sini San Ameliorates Duodenal Mucosal Barrier Injury and Low‑Grade Inflammation via the CRF Pathway in a Rat Model of Functional Dyspepsia.” International Journal of Molecular Medicine 45, no. 1: 53–60. 31746413 10.3892/ijmm.2019.4394 PMC6889936

[brb371135-bib-0080] Zou, X. , Y. Wang , Y. Wang , J. Yang , H. Guo , and Z. Cai . 2020. “Paeoniflorin Alleviates Abnormalities in Rats With Functional Dyspepsia by Stimulating the Release of Acetylcholine.” Drug Design, Development and Therapy 14: 5623–5632. 10.2147/DDDT.S260703. 33376306 PMC7764555

